# Receiving of emotional signal of pain from conspecifics in laboratory rats

**DOI:** 10.1098/rsos.140381

**Published:** 2015-04-01

**Authors:** Satoshi F. Nakashima, Masatoshi Ukezono, Hiroshi Nishida, Ryunosuke Sudo, Yuji Takano

**Affiliations:** 1 Human Information Science Laboratory, NTT Communication Science Laboratories, 3-1, Morinosato Wakamiya, Atsugi-shi, Kanagawa 243-0198, Japan; 2 CREST, Japan Science and Technology Agency (JST), 3-1, Morinosato Wakamiya, Atsugi-shi, Kanagawa 243-0198, Japan; 3 Department of Psychology, Meiji Gakuin University, 1-2-37, Shirokanedai, Minato-ku, Tokyo 108-8636, Japan; 4 Graduate School of Systems Life Sciences, Kyushu University, 6-10-1 Hakozaki, Higashi-ku, Fukuoka 812-8581, Japan; 5 Japan Society for the Promotion of Science, 5-3-1, Koji-machi, Chiyoda-ku, Tokyo 102-0083, Japan

**Keywords:** facial expression, pain, avoidance behaviour, laboratory rat

## Abstract

Though recent studies have shown that rodents express emotions with their face, whether emotional expression in rodents has a communicative function between conspecifics is still unclear. Here, we demonstrate the ability of visual recognition of emotional expressions in laboratory rats. We found that Long-Evans rats avoid images of pain expressions of conspecifics but not those of neutral expressions. The results indicate that rats use visual emotional signals from conspecifics to adjust their behaviour in an environment to avoid a potentially dangerous place. Therefore, emotional expression in rodents, rather than just a mere ‘expression’ of emotional states, might have a communicative function.

## 2. Introduction

The ability of conspecifics to discriminate emotional signals is a crucial skill in social animals. In humans, emotional facial expressions are well-known social signals for conveying emotional states [1] or the intention [2] of the expresser to other individuals. In the major emotion theory, emotional expression is considered to be an innate and universal mechanism. Cross-cultural studies have shown that recognition of emotional expression is culturally universal [3,4], although some cultural-specific display and decoding rules exist [5,6].

In another line of study, emotional expressions are considered to have the function of regulating internal physiological states, such as augmenting or diminishing exposure to environmental stimulation [7]. For instance, an expression of fear is adaptive for the detection of threatening stimuli in environments. One study has shown that the widened eyes in a fear expression increase the scope of the expressers' visual field and speed of eye movement [8].

Darwin argued that non-human animals can also express emotions with their face and body [9]. In fact, researchers have found that several kinds of primates show facial expressions in a regular way [10] and can discriminate categories of facial expressions [11]. For non-primate animals, two studies have shown that rodents are capable of showing facial emotional expression of pain [12,13]. In one, researchers developed a coding system for assessing facial expression of pain in mice [12].

Although previous studies have shown the presence of emotional expressions in rodents, to our knowledge no study has examined whether these emotional expressions have a communicative function or are mere ‘expressions’ as a by-product derived from the function of physiological regulation. The aim of the present study was to examine whether rodents use emotional expressions of conspecifics as social signals to adjust their behaviour in an environment through visual information. The emotional expression of pain might have an adaptive function for both the expresser and receiver, such as emitting a warning signal or promoting carer behaviour from the receiver [14]. If the emotional expression of pain has a communicative function, we predicted that rodents would be able to discriminate emotional expressions of pain from neutral expressions.

**Figure 1.**
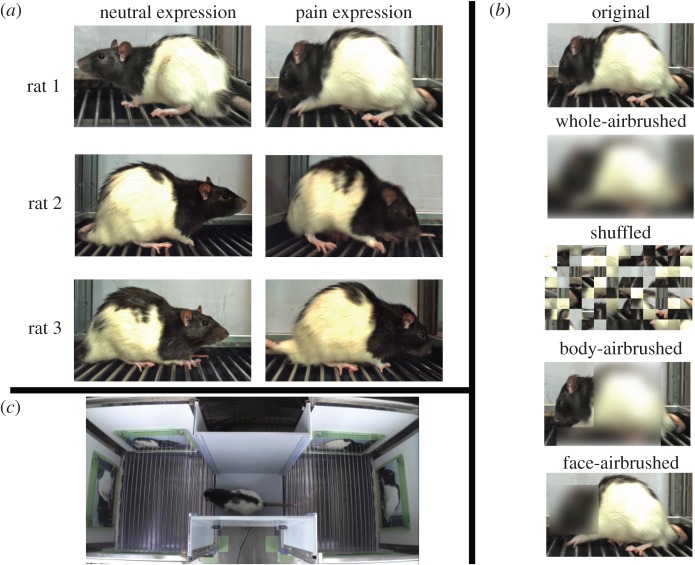
Image stimuli and apparatus used for preference test in rats. (*a*) Original photographs of pain and neutral expressions taken of three model rats. (*b*) Five categories of images used for preference test: original, whole-airbrushed, shuffled, body-airbrushed and face-airbrushed images. (*c*) The apparatus consisted of two side compartments and a central zone. Three photographs of individuals that showed either pain or neutral expressions were put in each side compartment.

We photographed three Long-Evans rats (not used in the main experiments) with painful or neutral facial expressions and prepared five categories of photographs of pain and neutral expression pairs: original image, all-airbrushed image, shuffled image, body-airbrushed image and face-airbrushed image (figure 1*a*,*b*). We created all-airbrushed images and shuffled images to determine whether luminance or other visual characteristics unrelated to emotional expression in the original images produce the effect of emotional expression. We created the face-airbrushed and body-airbrushed images to examine which part of the image is crucial for the discrimination of emotional expression.

The experimental apparatus consisted of a right side compartment, a left side one and a central zone [15]. The entrances of the right and left compartments were connected to the central zone. We attached photographs of the three individuals with pain expression on each wall of one of the side compartments and photographs of the neutral expressions on each wall of the other side compartment (figure 1*c*).

## 3. Experiment 1

### 3.1 Methods

#### 3.1.1 Animals and housing

A 104 naive male Long-Evans rats (Japan SLC, Inc.) weighing 230–340 g (8–12 weeks old) at the time of the experiment were pair-housed throughout the period of acclimation and experiment. Each pair was housed in a polycarbonate cage (26 cm width × 43 cm depth × 20 cm height). Approximately 36 g of standard laboratory food (CE-2, CLEA Japan, Inc.) was given to each cage per day. Rats had free access to tap water in their home cage. Temperature and humidity of the environment were controlled as constant (23±1^°^C and 50±5%, respectively) by a rearing system (EBAC-L, CLEA). The inside of the system was illuminated from 8.00 to 20.00.

#### 3.1.2 Pictorial stimuli

All images used in the experiment are shown in figure 1. We took the photographs by putting three rats in quadrangular operant test chamber (30 cm width × 30 cm depth × 63 cm height) with sliding clear plastic walls. The grid floor consisted of 19 stainless steel bars wired to an animal shocker (SGA2010, O'Hara & Co., Ltd). A high-speed camera (FL3-U3–13E4C-C, Point grey) was placed on one side of the apparatus. The high-speed camera can take 60 frames per second automatically. To take clear images, the plastic wall on the side of the high-speed camera was raised enough to be outside of the scope of camera. For each photographic subject, we took photographs of neutral expressions before taking those of pain expressions. After putting a rat on the floor of the chamber, we let the rat acclimate to the chamber for 5 min and then we took a number of photographs of the neutral expression of the rat. For the pain expression, we took a number of photographs of the rat while inflicting an approximately 0.5 s foot shock (2 mA) to the rat.

We selected photographs of pain expressions to use in the experiment on the basis of the norm in previous studies: tightening of the orbital region of the face, flattening of the ears and swelling of the nose and cheeks [12,13]. The original photographs were trimmed to make each photographic subject approximately the same size with respect to the width of the neck and the length of the ear. The photographs were 17.8 cm wide and 10.2 cm high.

We manipulated each photograph to create airbrushed stimuli using Photoshop Elements (Adobe Photoshop Elements 10, Adobe system). For all-airbrushed images, Gaussian blur with a radius of 50 pixels was applied on the whole photograph. For face-airbrushed images and body-airbrushed images, the same Gaussian blur was applied around the face (5.4–6.2 cm width × 5.4–6.2 cm height) and over the whole body (7.3–8.2 cm width × 9.1–10.2 cm height and 2.3–5.5 cm width × 1.7–2.8 cm height). We made shuffled stimuli using MatLab (Mathworks, Natick, MA, USA) as follows. Each original photograph was divided into 12 (horizontal) × 8 (vertical) segments (106×92 pixels; redundant pixels were eliminated). We then converged the segments after pseudo-randomly shuffling the order of the segments.

#### 3.1.3 Apparatus

In this study, we used an apparatus commonly used for measuring a conditioned place preference task [15]. The apparatus consisted of three compartments: two side compartments (30 cm width × 30 cm depth × 40 cm height), each with a grid floor and painted white, and a centre zone (20 cm width × 20 cm depth × 40 cm height) with a plain floor and painted grey. The entrance of each side compartment was connected to the centre zone. The luminance at the centre of each side compartment was approximately 103 lux. We put photographs of the three individuals with a pain expression on each wall of one side compartment and with a neutral expression on the each wall of the other side compartment. The location of the compartment with the photographs of pain or neutral expressions was counterbalanced between subjects. As the walls, floor, and luminance of each side compartment were approximately identical, we were able to assess the behaviour of subjects solely on the basis of the difference in the category of the photograph in each compartment.

#### 3.1.4 Preference test

Each of the animals in each group—original, whole-airbrushed, shuffled, body-airbrushed and face-airbrushed—was used in one experimental condition only. Each group comprised 21 animals with the exception of the shuffled group (20 animals). The procedure for each group was the same, except that the photographic image on the wall of the side compartments was different. For example, for the all-airbrushed group, the all-airbrushed photographic images of the pain and neutral expression were attached to the wall of each side compartment.

In the experiment, we placed each subject in the centre zone and left him to explore the apparatus for 10 min and recorded the time spent in and the frequency of entering each compartment. We started to record the staying time when both back paws of the rats had reached the entrance of a compartment.

### 3.2 Results and discussion

**Figure 2.**
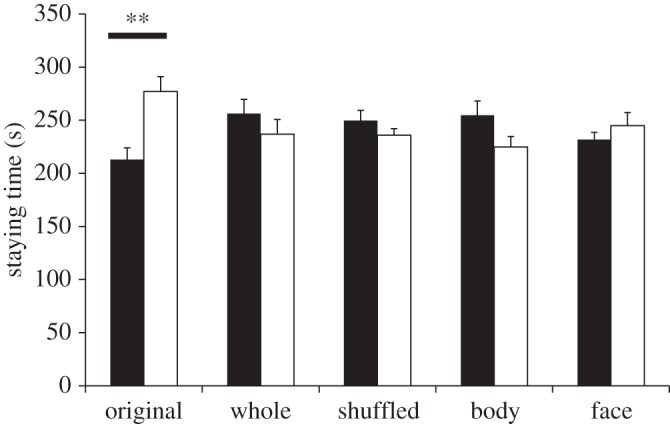
Mean time spent in the compartment with images of either pain or neutral expressions of conspecifics. In the original condition, rats stayed longer in the compartment with images of neutral expressions than in that with pain expressions. In the three airbrushed image conditions and shuffled image condition, there were no differences in the time spent in the compartments with the images of pain expressions and neutral expressions. Black bars indicate averaged staying time for pain expression; white bars indicate averaged staying time for neutral expression. Error bars represent s.e.m. ^**^p<0.01.

Figure 2 shows the time spent in each compartment with the images of pain and neutral expressions in each condition. We conducted a 5 (Condition) × 2 (Expression: neutral, pain) mixed factorial ANOVA for the length of time the rats spent in each compartment, which showed no main effects for either condition (*F*_4,99_=0.78, *p*=0.54, $\eta_{p}^{2}=0.03$) or expression (*F*_1,99_=0.08, *p*=0.77, $\eta_{p}^{2}<0.001$). By contrast, the results of the ANOVA showed significant interaction between condition and expression (*F*_4,99_=2.96, *p*=0.024, $\eta_{p}^{2}=0.11$). Subsequent analyses showed that the simple main effect of expression in the original image condition was significant (*F*_1,99_=8.46, *p*=0.005, $\eta_{p}^{2}=0.08$). In the original image condition, the rats stayed longer in the compartment with the images of neutral expressions than in that with the images of pain expressions. This indicates that the rats were able to discriminate the category of emotional expression.

On the other hand, we did not find any significant simple main effect of expression in the remaining conditions. The difference in the time spent in each compartment in the original image condition cannot be attributed to differences in mere visual characteristics between the images of emotional expressions because the rats spent the same amount of time in each compartment in the whole-airbrushed image condition (*F*_1,99_=0.78, *p*=0.38, $\eta_{p}^{2}=0.008$) and shuffled image condition (*F*_1,99_=0.39, *p*=0.53, $\eta_{p}^{2}=0.004$).

To examine which part of the images is crucial for rats to discriminate emotional expressions, we compared the time spent in each compartment in the body-airbrushed image and face-airbrushed image conditions. However, we did not find a significant difference in the time spent in each compartment (*F*_1,99_=1.83, *p*=0.18, $\eta_{p}^{2}=0.02$ for body-airbrushed and $F_{1,99}=0.36,p=0.55,\eta_{p}^{2}=0.004$ for face-airbrushed). The results indicate that the rats were unable to discriminate emotional expressions when the face or body was airbrushed.

**Figure 3.**
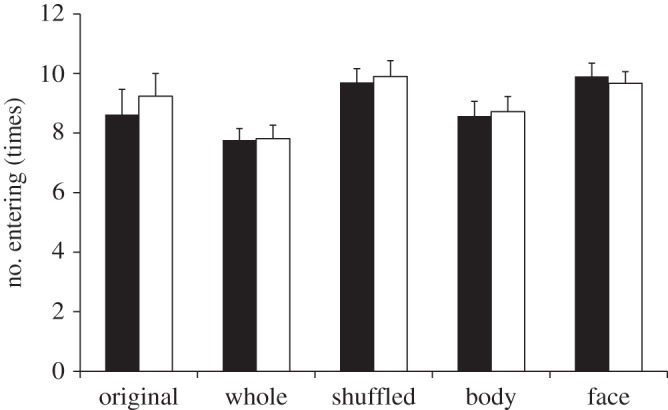
Mean frequency of rats entering each compartment with images of either pain or neutral expression in original image, whole-airbrushed image, shuffled image, body-airbrushed image and face-airbrushed image conditions. There were no differences in the frequency of entering between pain and neutral expressions in all conditions. Black bars indicate averaged staying time for pain expression; white bars indicate averaged staying time for neutral expression. Error bars represent s.e.m.

Compared to other conditions, the rats may have stayed in the compartment with the neutral face longer in the original condition because they had become frozen with anxiety. To test this possibility, we counted the number of times that rats entered each compartment (figure 3). We conducted a 5 (Condition) × 2 (Expression: neutral, pain) mixed factorial ANOVA of the number of times rats entered. Although the main effect of condition was marginally significant (*F*_4,99_ = 2.45, *p* = 0.051, $\eta_{p}^{2}\;=\;0.09$), a post hoc Bonferroni corrected *t*-test did not reveal any significant difference between each condition (all *p*s > 0.05). We did not find a significant main effect of expression (*F*_1,99_ = 1.15, *p* = 0.28, $\eta_{p}^{2}<0.01$) or interaction (*F*_4,99_ = 0.93, *p* = 0.45, $\eta_{p}^{2}=0.04$). The results indicate that the freezing behaviour is not the reason the rats stayed in the compartment with neutral faces longer in the original condition.

## 4. Experiment 2

In experiment 1, we did not habituate the rats to the experimental apparatus before measuring their behaviour in the preference test by reason of the possibility that rats might not be sensitive to detect pain expression of other conspecifics and/or might not show their natural behaviour to pain expressions. This is because they might learn that the inside of the boxes is safe if they are habituated to the boxes, though experimental animals are normally habituated to the apparatus before testing with a place preference task. We therefore conducted an additional experiment to examine whether the presence or absence of habituation to the experimental apparatus before testing affects the behaviour of perceiver rats to emotional expressions of conspecifics and to see if we could replicate the main findings of experiment 1 after habituation.

### 4.1 Methods

The experimental setting was same as the original condition in experiment 1 except that we habituated all animals to the experimental apparatus for 10 min 1 day before the experiment and put a mesh plastic mat [30 cm width × 30 cm depth] on the floor of each compartment to cover the grid throughout the experiment. We used 26 naive male Long-Evans rats (Japan SLC, Inc.) weighing 270–310 g (nine-weeks old) at the time of experiment. The location of the compartment with photographs of pain or neutral expressions was counterbalanced between subjects according to the time spent in each compartment during the habituation phase with time spent to each assigned compartment equalized (prospective pain side: *M*=246.69 s, s.d. = 40.16; prospective neutral side: *M*=252.35 s, s.d. = 43.70).

### 4.2 Results and discussion

**Figure 4.**
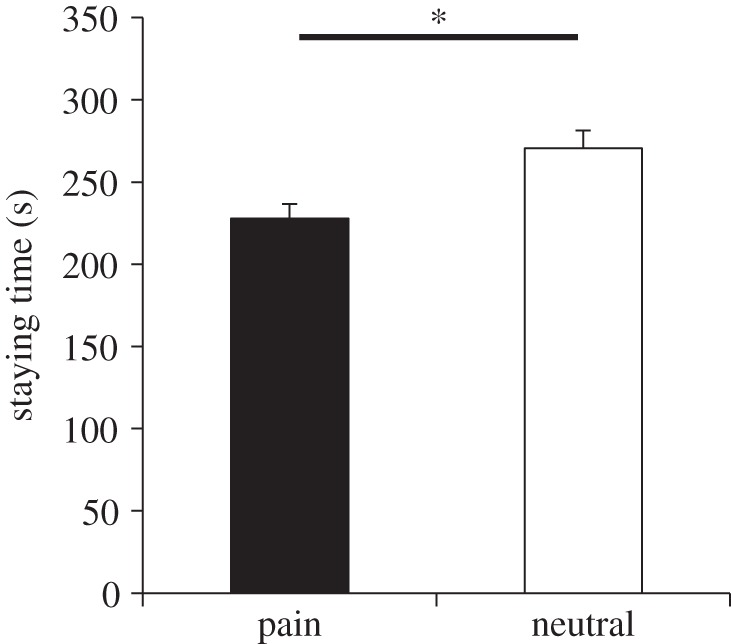
Mean time spent in the compartment with images of either pain or neutral expressions of conspecifics during the test phase after habituating rats to the environment without images. The rats stayed longer in the compartment with images of neutral expressions than in that with pain expressions. Black bars indicate averaged staying time for pain expression; white bars indicate averaged staying time for neutral expression. Error bars represent s.e.m. *p<0.05.

Figure 4 shows the time spent in each compartment with the images of pain and neutral expressions. As in the original condition in experiment 1, we found that rats stayed longer in the compartment with the images of neutral expressions than in the one with the images of pain expressions (*t*_25_=−2.25, *p*=0.03, *d*=0.45), which replicates the main findings of the first experiment. These results mean that the rats are able to discriminate categories of emotion and avoid pain expressions of other conspecifics, regardless of the presence or absence of habituation to the experimental environment.

## 5. General discussion

Previous studies have shown that rodents show emotional expressions [12,13]. However, whether rodents can use emotional expressions of conspecifics as a social signal was unknown. In general, the sensation modalities explored in research on communication in rodents have mainly been olfactory, pheromonal signal [16] and ultrasonic vocalization [17,18]; research on the visual modality has mostly been neglected [19].

However, there is evidence of the availability of visual information for social interaction in rodents. A study that examined social modulation of pain in mice by exposure to their cagemates has demonstrated that socially mediated hyperalgesia can be attributed to visual information from other individuals [20]. Therefore, visual cues might be crucial for receiving social signals from other individuals even in rodents. In line with this notion, our findings confirm that rats can discriminate emotional expressions of pain from neutral expressions in other individuals using only visual cues. Moreover, the result for the all-airbrushed condition and shuffled condition means that simple visual characteristics, such as the luminance or colour of pictorial stimuli, cannot explain our results.

We demonstrated that rats tend to avoid pain expressions of other individuals. This result looks incompatible with an earlier study that showed that mice tend to approach other mice that have been injected with a nociceptive stimulus [21]. However, the difference in the results might be due to the type of pain provided to the stimulus animals. Whereas, the previous study used intraperitoneal injection of acetic acid to prompt internal physiologically driven pain states in the stimulus animal, we used electrical foot shocks to induce a pain response to an exogenous harmful stimulus. In the former case, caring behaviour towards the stimulus animal might be adaptive because of the absence of potential danger to the carer; in the latter case, caring behaviour might carry a high risk to the carer. Rodents might discriminate the type of pain expression and alter their behaviour appropriately on the basis of the presence of the risk associated with pain expressions of conspecifics.

A remaining issue is which part of the photograph of emotional expression is essential for receiving social signals in rats. We did not find any significant difference between pain and neutral expressions in the face-airbrushed and body-airbrushed conditions. One possible explanation is that the rats did not perceive the presence of other individuals in either condition because the airbrushing created an unnaturalness that does not exist in the rat's social world.

Our study might contribute to exploring the process of evolution of emotional expression. Whether emotional expression in rodents has a communicative function or is a mere ‘expression’ has been controversial. From evolutionary perspectives, it has been hypothesized that the original function of emotion expression was to regulate the internal physiological states of organisms to respond adaptively to environmental stimuli and, in later stages of evolution, the communicative function of emotional expression was acquired [7]. Our results indicate that emotional expression in rodents, rather than just a mere ‘expression’ of emotional states, might have a communicative function.

However, we should mention that there is still a remaining issue concerning the interpretations of the results of the study. Although we showed that ‘perceiver’ rats could discriminate emotional expressions of other individuals, it is still unclear whether they actually recognized that conspecifics were in pain or not. Another possible interpretation of why the rats avoided pain expressions would be simple association learning between pain expression and aversive experience. For instance, perceiver rats might which see pain expressions of other individuals while simultaneously experiencing an aversive event might automatically associate the pain expressions with the aversive event. In this case, rats could avoid pain expressions of other individuals without inferring the emotional states of others. In this regard, however, one should recall that the experimental animals in the study were all naive rats. In other words, we never gave an aversive electrical shock to perceiver rats along with pain expressions of other rats during the feeding and experimental periods. They therefore did not have an opportunity to learn the association between pain expression and negative experience, at least not in an explicit manner, though we cannot deny the possibility that they learned such an association with their cagemate during daily life. Further studies are needed in order to examine such a possibility.

In conclusion, we showed the ability of visual recognition of facial expressions in laboratory rats. Our technique for measuring the recognition of emotional expressions in rats will be useful in neuroscientific research for exploring neural mechanisms of recognition of emotional expressions in rodents. The experimental technique is advantageous for examining visual processing in rodents because it excludes the influence of other sensory information.

## Data accessibility

Raw dataset is available on Datadryad.org ().
